# Supplementary material for: Transcriptomic Insights into Mechanisms of Early Seed Maturation in the Garden Pea (Pisum sativum L.)
Source: Cells. 2020 Mar 23;9(3):779. doi: 10.3390/cells9030779 (PMC7140803; doi:10.3390/cells9030779)
Supplement: Supplementary file 1 [file cells-09-00779-s001.zip › Supplementary materials/FileS2.zip!/FileS1/Summary Sheets - Modules/K-Means Cluster/Profiles.pdf]

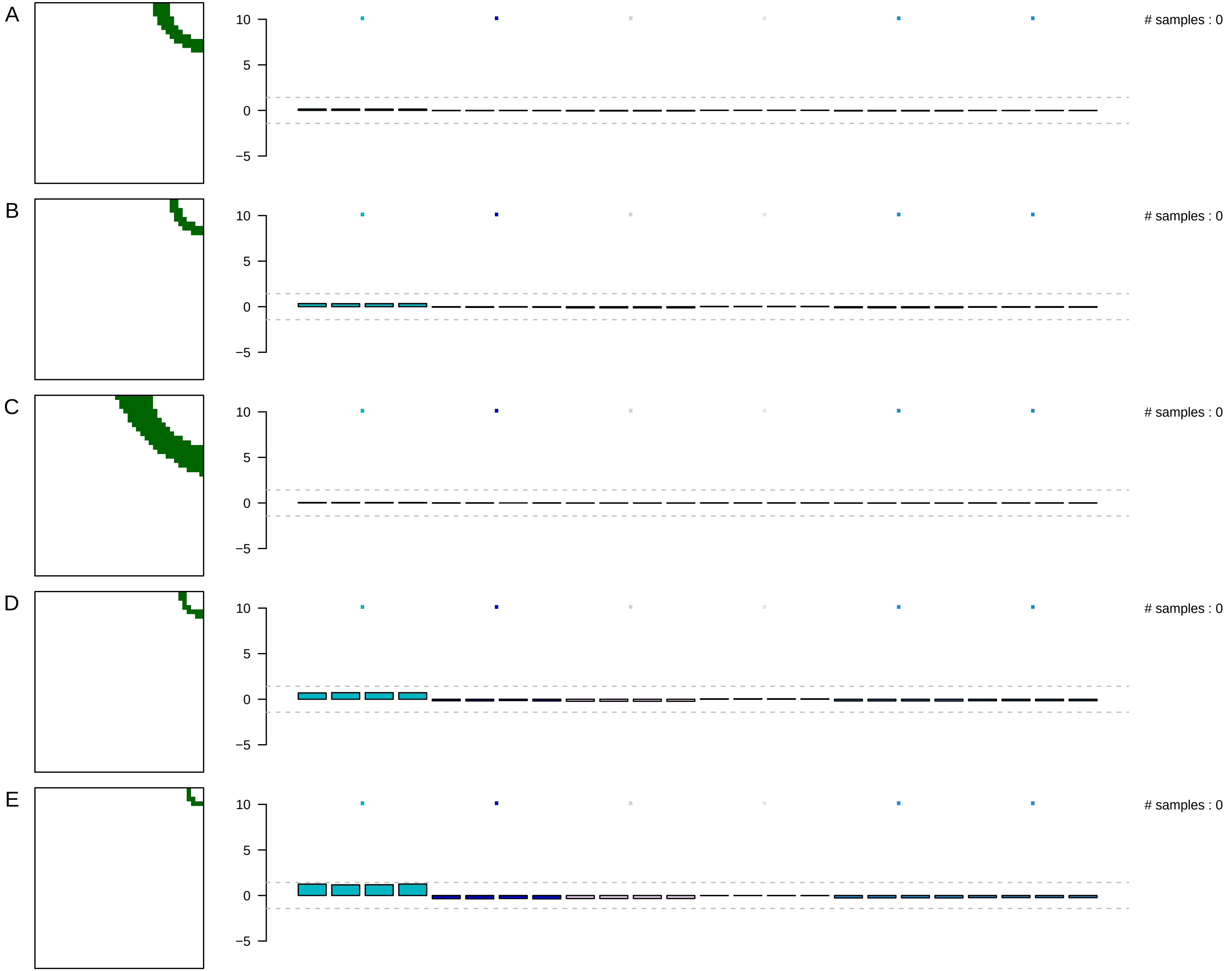
A
B
C
D
E
10
5
0
−5
samples : 0

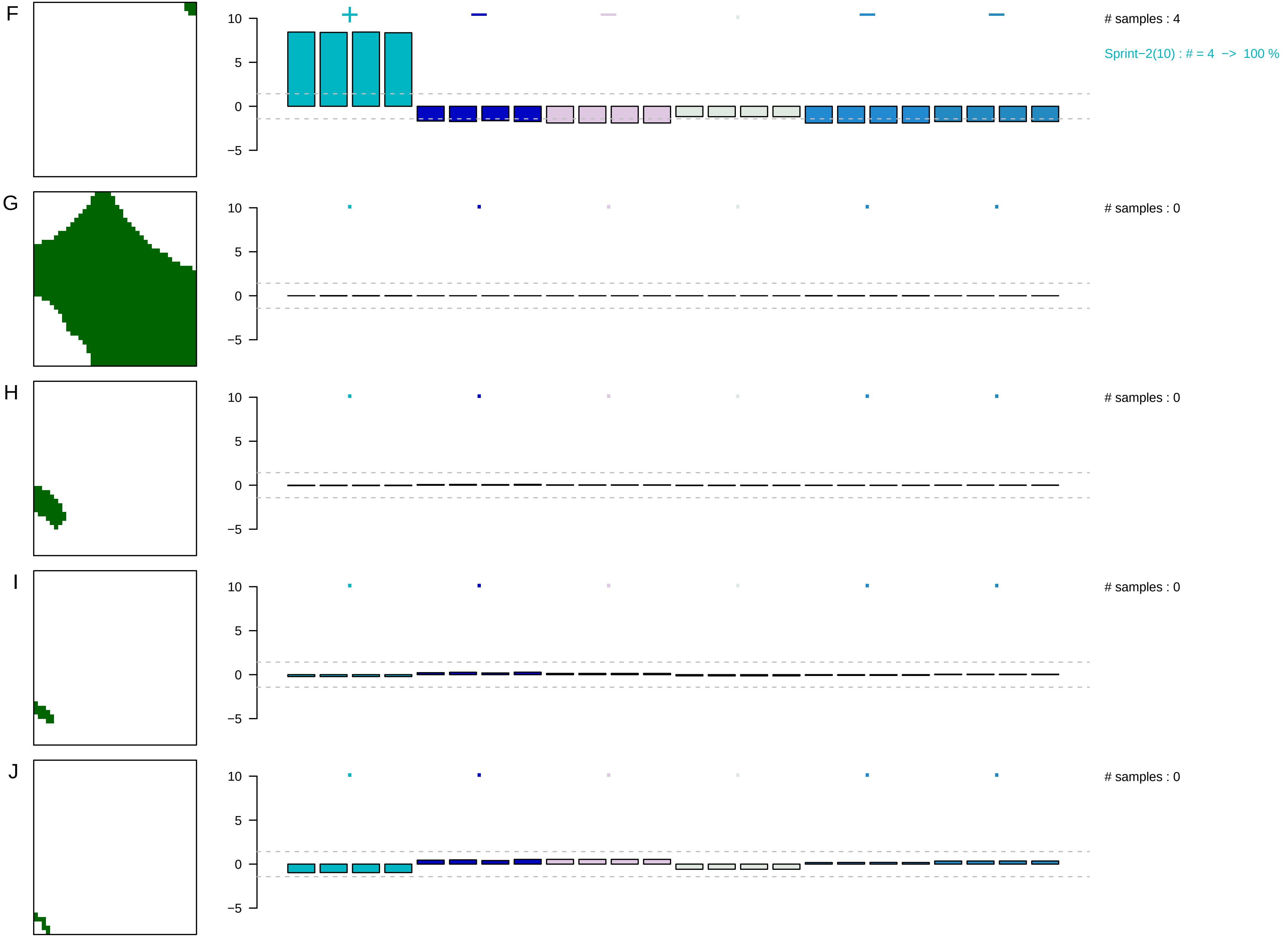
F
samples : 4
Sprint−2(10) : # = 4 −> 100 %
G
samples : 0
H
samples : 0
I
samples : 0
J
samples : 0
10
5
0
−5

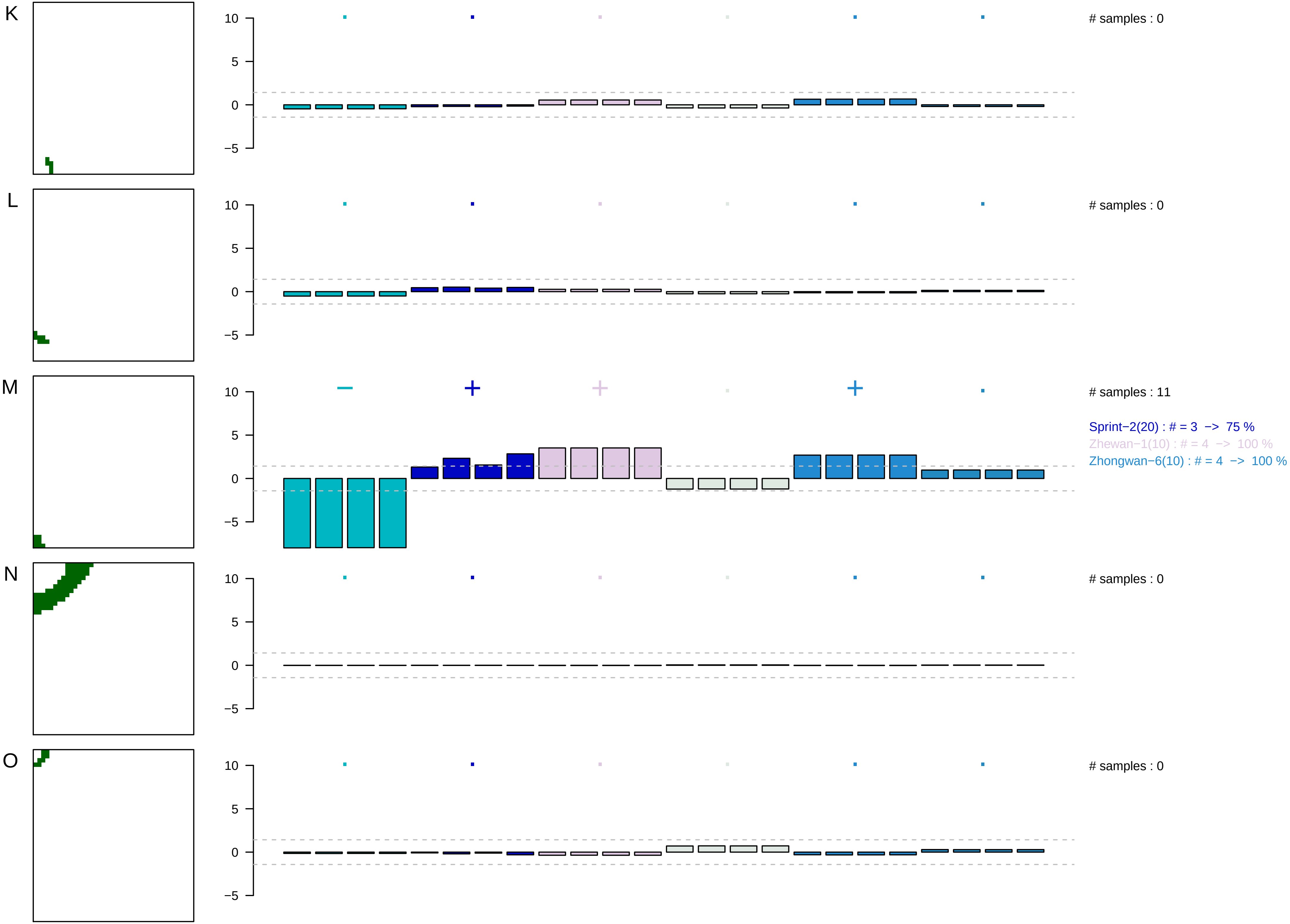
K
L
M
N
O
10
5
0
−5
samples : 0
samples : 0
samples : 11
Sprint−2(20) : # = 3 −> 75 %
Zhewan−1(10) : # = 4 −> 100 %
Zhongwan−6(10) : # = 4 −> 100 %
samples : 0
samples : 0

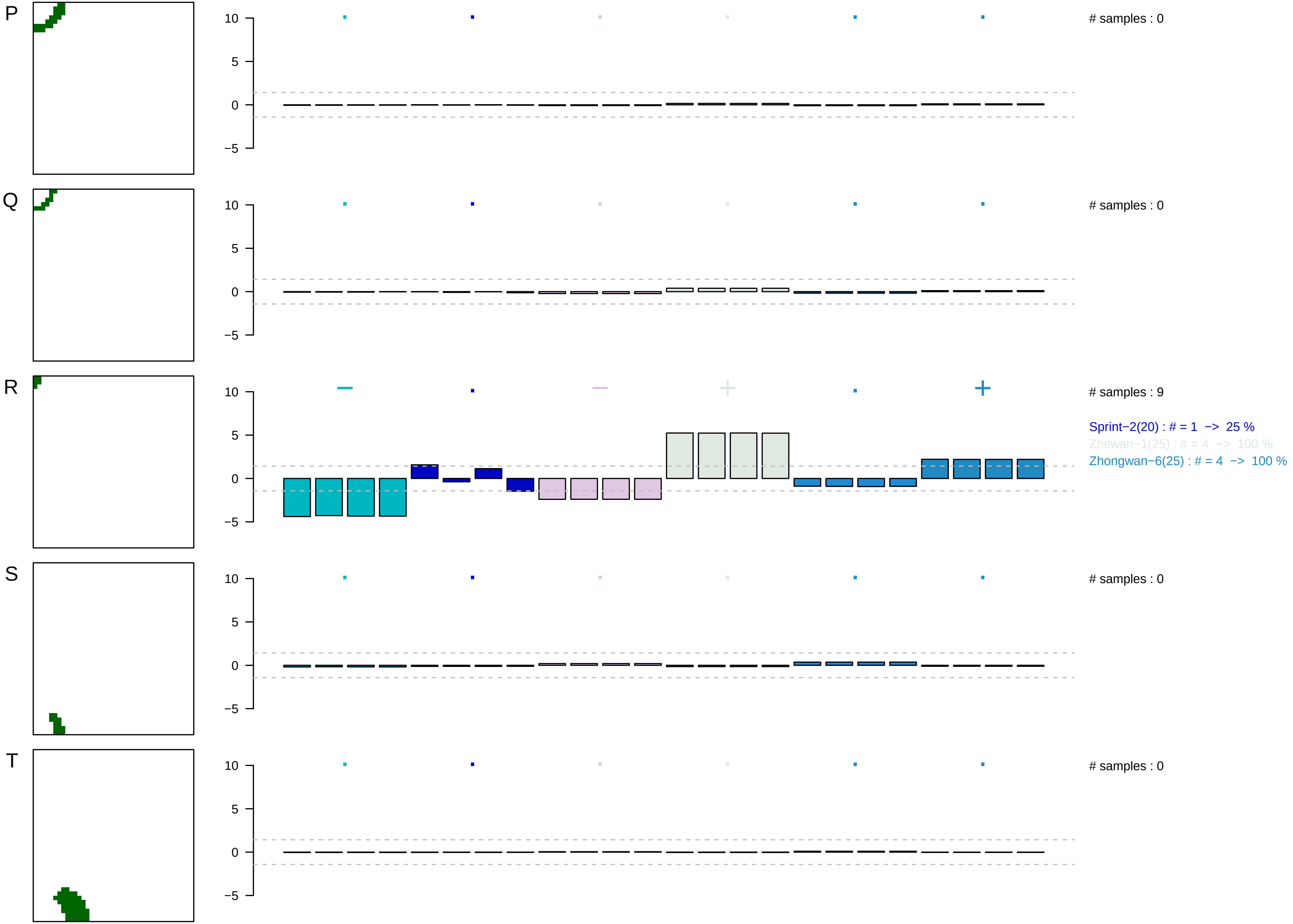
P
samples : 0
Q
samples : 0
R
samples : 9
Sprint−2(20) : # = 1 −> 25 %
Zhewan−1(25) : # = 4 −> 100 %
Zhongwan−6(25) : # = 4 −> 100 %
S
samples : 0
T
samples : 0
10
5
0
−5

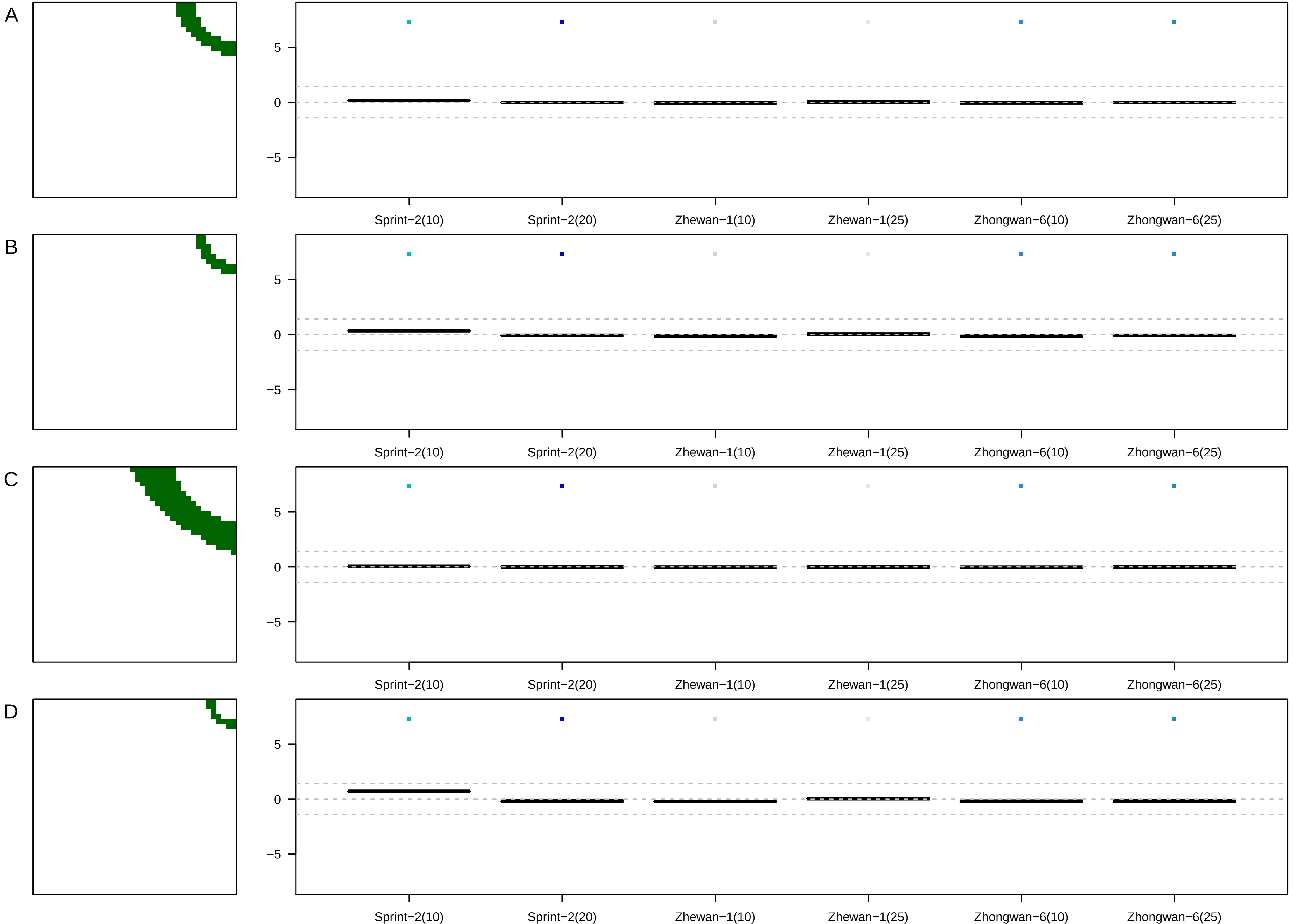
A
B
C
D
5
0
−5
Sprint−2(10)
Sprint−2(20)
Zhewan−1(10)
Zhewan−1(25)
Zhongwan−6(10)
Zhongwan−6(25)

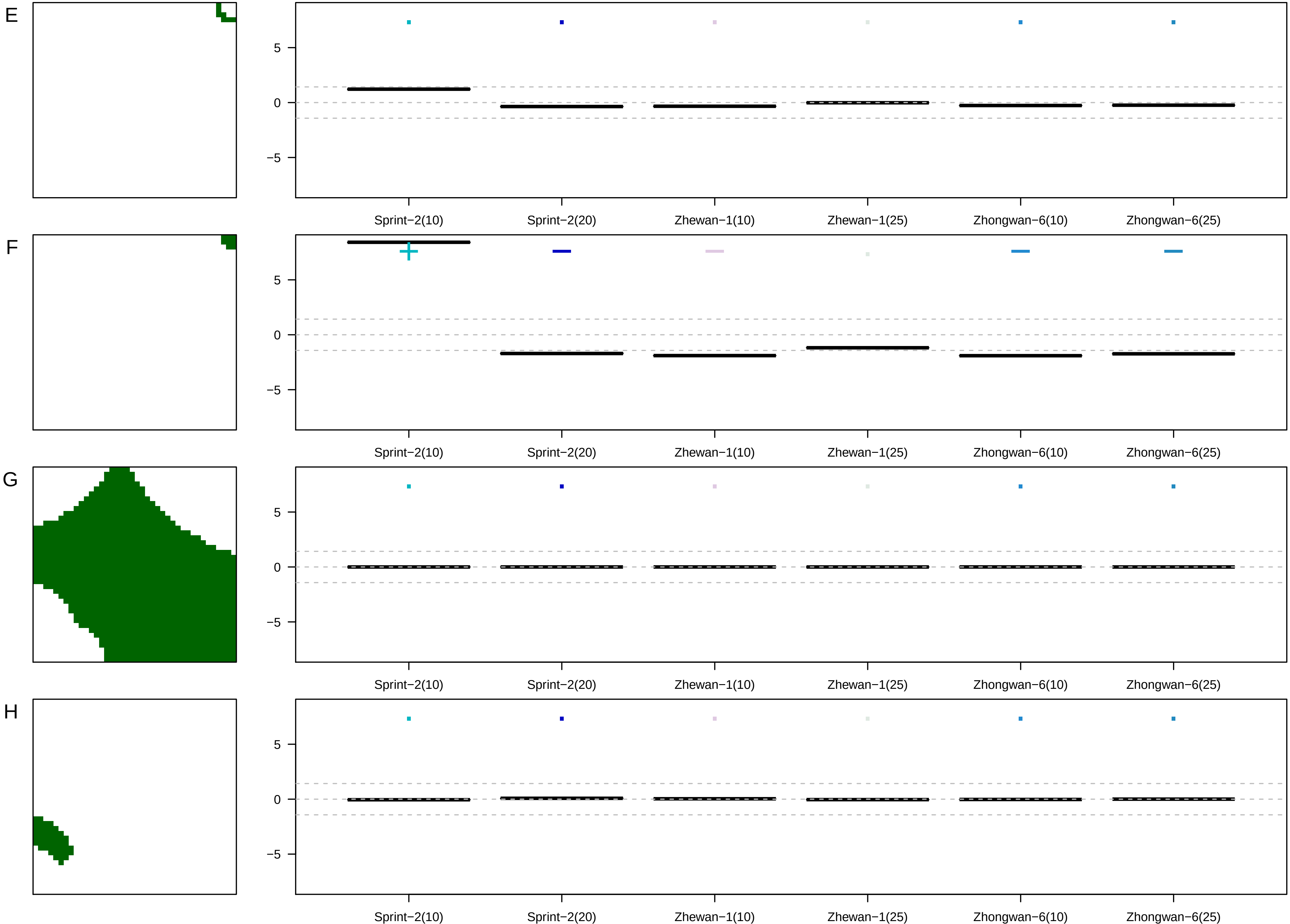
E
F
G
H
5
0
−5
Sprint−2(10)
Sprint−2(20)
Zhewan−1(10)
Zhewan−1(25)
Zhongwan−6(10)
Zhongwan−6(25)

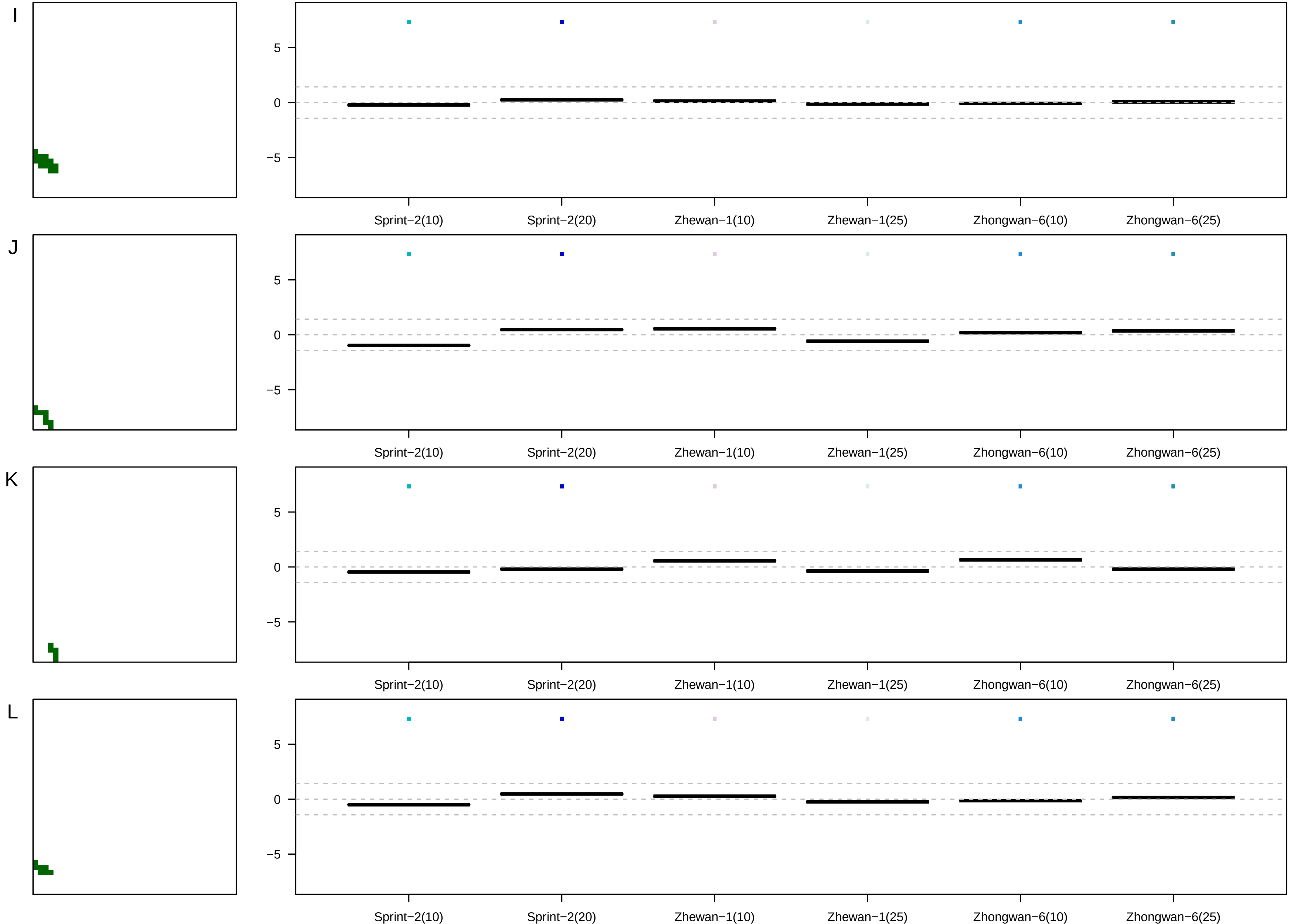
I
5
0
−5
Sprint−2(10)
Sprint−2(20)
Zhewan−1(10)
Zhewan−1(25)
Zhongwan−6(10)
Zhongwan−6(25)
J
5
0
−5
Sprint−2(10)
Sprint−2(20)
Zhewan−1(10)
Zhewan−1(25)
Zhongwan−6(10)
Zhongwan−6(25)
K
5
0
−5
Sprint−2(10)
Sprint−2(20)
Zhewan−1(10)
Zhewan−1(25)
Zhongwan−6(10)
Zhongwan−6(25)
L
5
0
−5
Sprint−2(10)
Sprint−2(20)
Zhewan−1(10)
Zhewan−1(25)
Zhongwan−6(10)
Zhongwan−6(25)

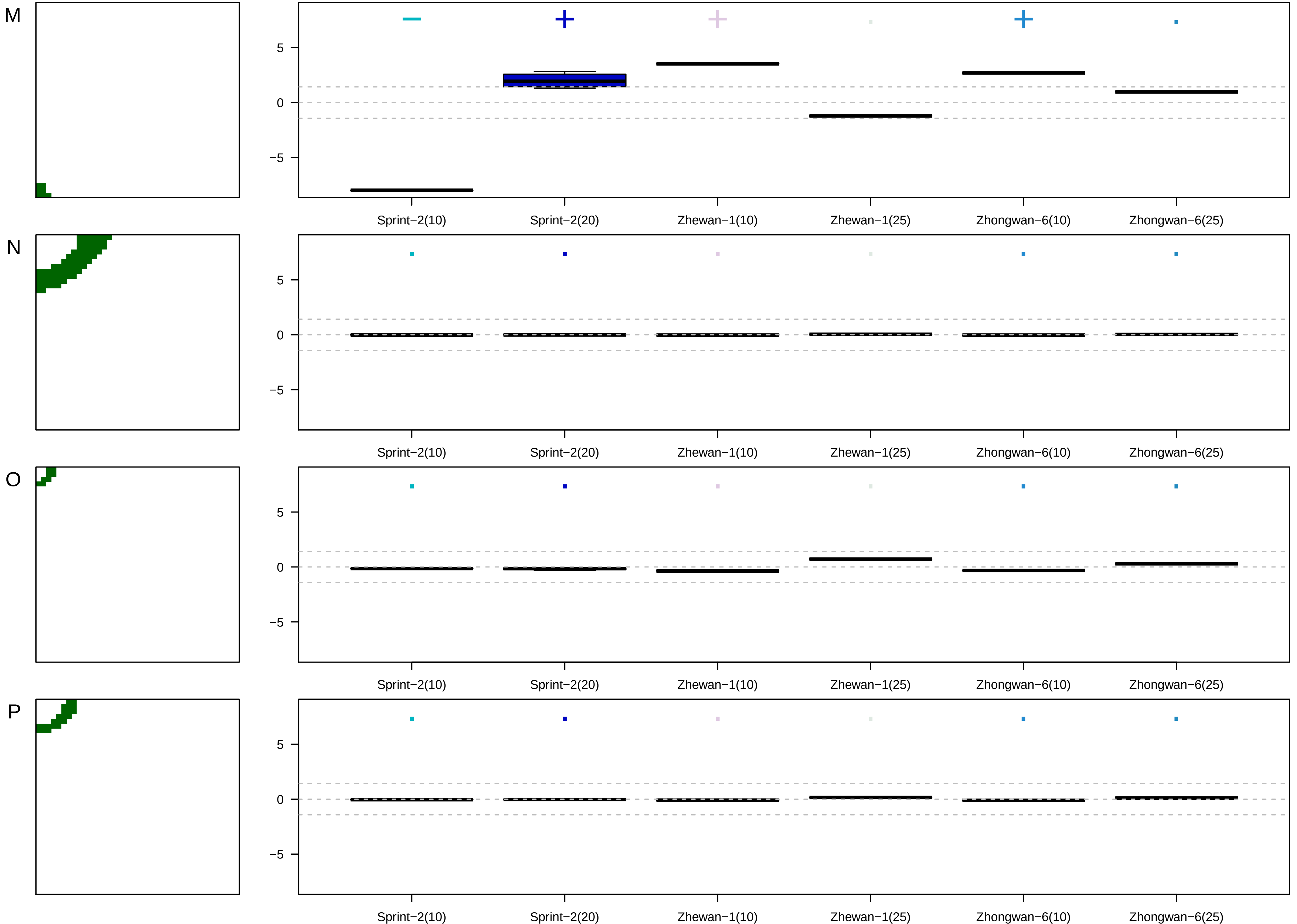
M
N
O
P
5
0
−5
Sprint−2(10)
Sprint−2(20)
Zhewan−1(10)
Zhewan−1(25)
Zhongwan−6(10)
Zhongwan−6(25)

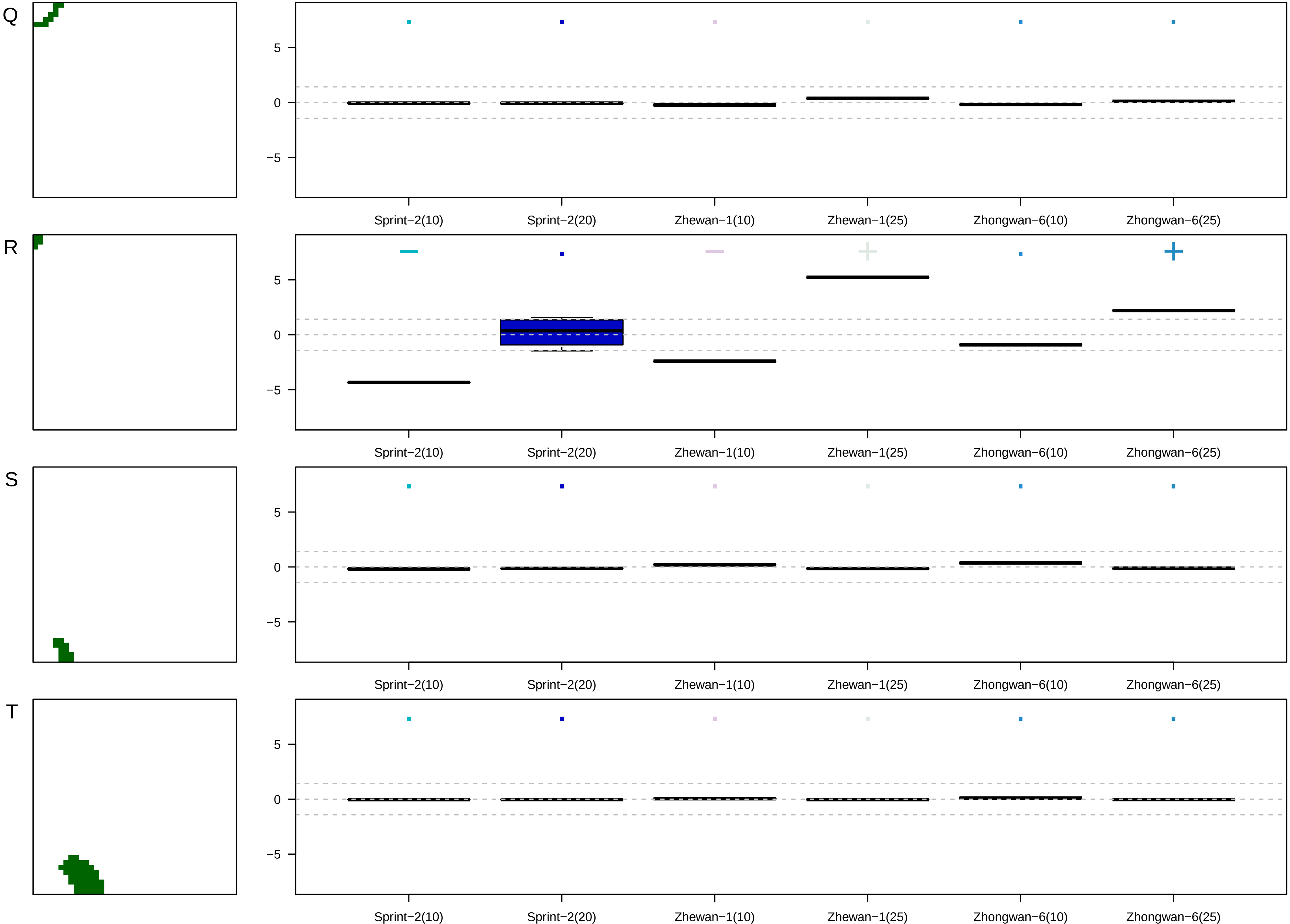
Q
5
0
−5
Sprint−2(10)
Sprint−2(20)
Zhewan−1(10)
Zhewan−1(25)
Zhongwan−6(10)
Zhongwan−6(25)
R
5
0
−5
Sprint−2(10)
Sprint−2(20)
Zhewan−1(10)
Zhewan−1(25)
Zhongwan−6(10)
Zhongwan−6(25)
S
5
0
−5
Sprint−2(10)
Sprint−2(20)
Zhewan−1(10)
Zhewan−1(25)
Zhongwan−6(10)
Zhongwan−6(25)
T
5
0
−5
Sprint−2(10)
Sprint−2(20)
Zhewan−1(10)
Zhewan−1(25)
Zhongwan−6(10)
Zhongwan−6(25)
